# Implementation, home mediators and children’s sugary drink consumption - results from DAGIS study

**DOI:** 10.1093/eurpub/ckac130.159

**Published:** 2022-10-25

**Authors:** R Lehto, H Vepsäläinen, A-V Lehtimäki, E Lehto, MH Leppänen, E Skaffari, A Abdollahi, E Roos, M Erkkola, C Ray

**Affiliations:** 1 Folkhälsan Research Center, Helsinki, Finland; 2 Department of Food and Nutrition, University of Helsinki, Helsinki, Finland; 3 Department of Sociology, University of Helsinki, Helsinki, Finland; 4 Faculty of Medicine, University of Helsinki, Helsinki, Finland; 5 Department of Food Studies, Nutrition and Dietetics, Uppsala University, Uppsala, Sweden

## Abstract

**Background and objectives:**

The effectiveness of a health behavior intervention can depend on the extent to which the intervention is implemented; higher degree of implementation (DOI) might associate with larger intervention effects. This study examined whether the parental DOI of an health behavior intervention had an effect on children's consumption of sugar-sweetened beverages (SSB) and was the effect mediated by home factors.

**Methods:**

the DAGIS preschool intervention was conducted in 2017-2018 in Finland among 3-6-year-olds with valid data from 476 children. At baseline and follow-up parents reported 1) children's SSB consumption in a semi-quantified food frequency questionnaire, 2) availability of SSB at home, parental role modelling of drinking SSB, and norm (parental view on the suitable amount of SSB for children), and 3) DOI: a dichotomized sum variable on several aspects of parental program implementation. In the analyses, high and low DOI were compared to control group. Mediation analysis of the effect of DOI on the change in children's SSB consumption via change in availability, role modelling and norm was conducted with R statistical software.

**Results:**

High DOI was associated with reduced consumption of SSB (B -27.71, 95% CI -49.05, -4.80). No mediated effects were found. All studied mediators impacted the change in SSB consumption, but the DOI had no effect on the change in mediators.

**Conclusions:**

Intervention effect on the consumption of SSB was only found in the high DOI group, which supports the importance of assessing intervention implementation. Since the found effect was not mediated by the studied mediators, other possible mediators should be examined, as understanding intervention mediators is crucial in developing successful interventions.

**Key messages:**

- The consumption of SSB reduced only among children whose parents implemented the intervention to a large degree.
- The effect was not mediated by availability of SSB, parental role modelling, or norm.

